# Risk factors associated with suicidal ideation and suicide attempts in Bhutan: An analysis of the 2014 Bhutan STEPS Survey data

**DOI:** 10.1371/journal.pone.0225888

**Published:** 2020-01-30

**Authors:** Tashi Dendup, Yun Zhao, Tandin Dorji, Sonam Phuntsho

**Affiliations:** 1 Population Wellbeing and Environment Research Lab, School of Health and Society, University of Wollongong, NSW, Australia; 2 School of Public Health, Faculty of Health Sciences, Curtin University, Perth, Western Australia; 3 Ministry of Health, Royal Government of Bhutan, Kawangjangsa, Thimphu, Bhutan; Chiba Daigaku, JAPAN

## Abstract

Suicide is a major public health problem globally. Data on the factors influencing suicidal behaviours that can inform prevention policies are limited in Bhutan. This study used the dataset of the nationally-representative Bhutan STEPS Survey conducted in 2014 that assessed the non-communicable disease risk factors. Using a backward elimination approach, multiple logistic regression analysis accounting for the complex survey design was performed to identify the factors associated with suicidal ideation and suicide attempts in adults separately. The prevalence of suicidal ideation and suicide attempt was 3.1% and 0.7%, respectively. We found female gender, being unemployed, low and middle household income than high household income, and having a family history of suicide were associated with higher odds of having suicidal ideation. Younger age and alcohol consumption were associated with both suicidal ideation and suicide attempts. While those from the middle-income group compared to those in the high-income group had reduced odds of attempting suicide. The findings can help inform policy investments for suicide prevention. Prevention programs that target young people, females, and low socioeconomic groups, and aimed to reduce harmful alcohol use can help prevent suicidal behaviours.

## Introduction

With more than 800,000 individuals dying by suicide and many attempting it every year, suicide is a major global public health problem [1]. The crude suicide rate in 2016 was estimated to be 10.6 per 100,000 population (13.5 among males and 7.7 among females) [2]. More than 75% of the global suicide deaths occur in low- and middle-income countries [1]. The occurrence of suicide increases during adolescence and continues to grow in early adulthood [3]. Suicide rates are found highest among older adults [4]. Suicidal ideation (also referred to as suicidal thoughts) means thinking about, considering, or planning suicide [4]. Suicide attempt (also known as failed or non-fatal attempt) is a non-fatal self-directed, potentially injurious behaviour with an intention to end life [4]. The global lifetime prevalence of suicidal ideation and suicide attempts are estimated at 9.2% and 2.7%, respectively [5]. Suicidal ideation and attempt are strong predictors of future suicide attempts and suicide deaths and can lead to adverse economic and health impacts such as injury and hospitalization [1, 5–7]. Identifying factors influencing suicidal thoughts and attempts can be, therefore, crucial in informing suicide prevention policies and interventions.

Numerous studies have examined the factors influencing suicidal behaviors that include suicidal ideation, plan, and attempts. Being female [3–5, 8–12], younger age [3–5, 8], unmarried [5, 8], unemployed [10], low education and income [3, 5, 8] were reported to be associated with suicidal behaviours. Mental ill-health [3–5, 8, 9], loneliness [3, 9], depression [4, 12], feeling hopeless and unhappy [3, 4, 11, 13–15], impulsivity [3, 4], conduct problems [4, 12], and adversities during childhood [3, 8] were also identified as important risk factors for suicidal ideation and attempts. Additionally, family history of suicide [16–18], alcohol use [11, 13–15, 19], overweight [12, 13], inadequate physical activity [13, 20], illicit drug use [4, 11], and smoking [7, 9, 11, 13] have been documented to predict suicidal behaviours. Other factors linked with suicidal behaviours include insufficient social support and connectedness [3, 9, 10], parental separation and inadequate parental attachment [3, 14, 21], unstable relationship [10], truancy [9, 15], sexual intercourse initiation [11, 13, 14], physical fight [11, 13, 15], and being attacked and insulted [3, 11].

Suicide is among the top leading causes of mortality in Bhutan [22]. The estimated age-standardized suicide rate was 17.8 per 100,000 population in 2012 [1], and the crude suicide rate in 2017 was 12 per 100,000 population [23]. The annual average suicide growth rate during the year 2009–2013 was reported to be as high as 9.4% [24]. The high rate can be partly attributable to the rapid societal transformation that includes rural-urban migration, changes in social norms such as a shift away from the traditional extended family system especially in urban areas, rise in crime rates, and increase in mental health conditions [25]. As per the 2014 Bhutan National Survey for Noncommunicable Disease (NCD) Risk Factors and Mental Health conducted using the World Health Organization (WHO) STEPwise surveillance approach (referred to as Bhutan STEPS Survey in the present study), the suicide ideation prevalence among adults was 2.4% [26]. The 2015 Bhutan Gross National Happiness (GNH) Survey showed a higher suicidal thoughts prevalence of 5% [27], suggesting a substantial increase over just two years, and both surveys found greater prevalence among females than males [26, 27]. Furthermore, these two surveys reported that around 1% ever attempted suicide with a higher proportion among young adults. The 2016 Bhutan Global School-Based Health Survey that recruited students aged 13–17 years also showed a higher prevalence of suicidal ideation (14%) and suicidal attempts (11%) among young adolescents [28]. Recognizing suicide as a significant health and social issue, the National Suicide Prevention Program was instituted under the Ministry of Health, Royal Government of Bhutan in 2014 [22, 25].

Suicide prevention in Bhutan is in its early stages. Until the institution of the standalone program, some activities aimed to address one or a few factors such as prevention of domestic violence, alcohol and illicit drug use, and mental health counselling were pursued by different agencies including non-governmental organizations [22]. Some of the current prevention efforts include awareness activities conducted in collaboration with sectors such as schools and religious institutions, the establishment of a 24-hour crisis support hotline, and training of health workers as first responders [25]. The awareness activities include awareness on parenting, substance abuse, and presentation of mental disorders, mainly aimed to identify those at high risk. Suicide has been included in the module for the training of primary healthcare workers, and there is an increased engagement of school peer counsellors and teachers, and non-governmental organizations [25]. A national suicide registry managed by the national program is also in place where all incidents of suicide and suicide attempts are recorded and reported. However, the need for a more structured approach towards prevention, enhancing institutional capacity, strengthening community systems, and better data for effective planning and delivery of prevention services has been acknowledged [25].

There is a paucity of data on factors influencing suicidal thoughts and behaviour in Bhutan [25]. The 2014 Bhutan Suicide Study that interviewed next of kin of those who died by suicide indicated that the majority of those who completed and attempted suicide were 15–40 years old; more males committed suicide, whereas more females attempted suicide [29]. In addition, the survey reported higher suicide rates among rural residents, farmers, followed by students, and those who were married or coupling, and had low education and income. Domestic violence, marital problems, academic pressure, stressful life events, distress, alcohol and illicit drug use, and feeling hopeless were also highlighted as possible factors. This survey was limited by the descriptive analysis and did not investigate factors influencing suicidal ideation using statistical regression analysis. A study that analysed the data from the 2015 GNH Survey showed that younger age (15–24 years), females, mental distress, poor family relationship, negative emotions, disability, poor physical health, and being a crime victim were associated with higher odds of suicidal thoughts and behaviour [23]. However, this study did not examine some important factors, such as the family history of suicide, alcohol use, body weight, physical activity, and smoking due to the non-availability of relevant information. These behavioural factors associated with urbanization and societal changes may not just contribute to the rising burden of chronic conditions such as diabetes and heart diseases but also to that of poor mental health. A better understanding of their role on different health conditions including suicidal behaviours can help inform cost-effective interventions. Therefore, we examined the factors associated with suicidal ideation and suicide attempts using the nationally-representative Bhutan STEPS Survey data. Our study extends prior works by including some of the important factors that were not assessed previously and by accounting for the complex survey design and potential confounding effect.

## Methods

### Study setting

Bhutan is a small mountainous country bordered by China in the north and India in the south. Bhutan is more recently known to the world for its development philosophy of Gross National Happiness [30, 31]. The population in 2018 was 727,145 [32], and the per capita Gross National Income in 2018 was estimated at USD 2,809 [33]. With an area of 38,394 sq.km, the country is administratively divided into 20 districts. Health services are provided free of charge at the point of delivery by the state through a three-tiered health care system that is built upon the primary health care principles. Presently, there are 211 Basic Health Units at the local level, 26 district-level hospitals, three regional referral hospitals, of which one also functions as the national referral hospital [34].

### Data source

This study used the data from the Bhutan STEPwise surveillance (STEPS) cross-sectional survey conducted in 2014 [26]. Detailed information on this survey is available in the 2014 Bhutan STEPS Survey report published previously [26]. Briefly, the survey provides information on NCD prevalence and modifiable risk factors for men and women between 18–69 years of age. The survey questionnaire was pilot-tested and validated, and the data collection occurred from April to June 2014. A multi-stage sampling approach was used to recruit the participants, and the survey achieved a response rate of 97%. The media campaign about the survey via television and radio, the involvement of community health workers, and the rigorous filed supervision might have enabled this high response rate. This survey provided information on some of the potential risk factors (such as family history of suicide, alcohol use, body weight, physical activity, and smoking) that were not collected in the 2015 Bhutan GNH Survey [23]. After excluding five observations without information on suicidal ideation and one without information on suicide attempts, the final sample for this study included 2,816 participants. Ethical clearance for this study was granted by the Bhutan Research Ethics Board of Health.

### Study variables

#### Dependent variables

To be used in the binary logistic regression analysis, the self-reported “Yes” responses to the questions, “During the past 12 months, have you seriously considered attempting suicide?” and “During the past 12 months, have you made a plan about how you would attempt suicide?” were extracted and coded as “1” for suicidal ideation. Similarly, “Yes” responses to the question “Have you ever attempted suicide?” were coded as “1” for having attempted suicide. For both outcome variables, “No” responses were coded “0”.

#### Independent variables

The selection of independent (explanatory) variables was informed by the data in the literature [3–5, 7–9, 12, 16, 19, 20, 23, 29] and the availability of information in the 2014 Bhutan STEPS Survey. The demographic variables included were age (categorized as 18–30, 31–40, 41–50, 51–69 years), gender (male, female), and marital status (single/previously married, married/cohabiting). Education level (no education, ≥primary schooling), employment status (employed, self-employed, unemployed/others), annual household income (divided into tertiles—lowest representing the poorest), and place of residence (urban, rural) were the socioeconomic factors. The behaviour and health variables comprised of smoking (non-smoker, ex-smoker, and current smoker), alcohol use (non-drinker, ex-drinker, current drinker (≤2 days per week), current drinker (>2 days per week)), moderate-vigorous physical activity (yes, no), body mass index (BMI—<25 kg/m^2^, ≥25 kg/m^2^), high blood sugar (yes, no), high blood pressure (yes, no), and family history of suicide (yes, no).

Responses to the questions, on “ever consumed any alcohol, consumed any alcohol and frequency of having at least one standard alcoholic drink in the past 12 months,” were used to define the alcohol use. Blood pressure, blood sugar levels, and BMI were assessed using physical and biochemical measures. In consistent with the Bhutan STEPS Survey report [26], those with a systolic blood pressure of ≥140mmHg and a diastolic blood pressure of ≥90mmHg were categorized as having high blood pressure. Similarly, high blood sugar level was defined using a cut-off of >110mg/dl. The WHO cut-point for overweight/obesity used in the STEPS Survey was adapted to categorize the BMI [35]. The variable moderate-vigorous physical activity was derived from the responses on engagement in moderate- and vigorous-intensity work-related, and sports, fitness, or recreational activities.

### Data analysis

Frequencies and descriptive statistics were used to describe the characteristics of the study population and compute the prevalence of suicidal ideation and suicide attempts by the demographic, socioeconomic, and behaviour and health variables. Chi-square test (or Fisher’s exact test as applicable) was used to assess the difference in the prevalence between the groups of interest. The unadjusted associations between each explanatory variable and suicidal ideation and suicide attempts were assessed initially using simple logistic regression analysis. Those explanatory variables found significant at 10% level in the simple logistic regression analysis were then included in the multivariable regression model to identify factors independently influencing the odds of having suicidal ideation and suicide attempts, using a backward elimination approach. We also included some factors such as alcohol use and place of residence in the final model to reassess their effect. The results were presented as crude and adjusted odds ratios along with 95% confidence intervals. A p-value of <0.05 was considered statistically significant in the final model. We used the STATA survey (svy) command to analyse the data accounting for the complex survey design effect, including stratification, clustering, and sampling weights used in the Bhutan STEPS Survey. The data analysis was carried out in STATA version 14 package.

## Results

### Sample characteristics

The mean (SD) age of participants was 40.3 (12.55) years. Table 1 presents the characteristics of the study sample and the distribution of suicidal ideation and suicide attempts by the explanatory variables. The majority of the participants were >30 years of age (73.5%), females (62%), married (80.8%), had no formal education (62.6%), self-employed (55.3%), and lived in rural areas (69.2%). Most of the respondents never smoked (77.3%), were physically active (89.7%), did not have high blood sugar (90.1%), and did not report of suicide history in the family (97.5%). Around 37% of the participants were overweight or obese, 38.2% had high blood pressure, and 48.1% currently consumed alcohol.

**Table 1:** Characteristics of the participants and the distribution of suicidal ideation and suicide attempts by these characteristics (N = 2,816).

Variables		Suicidal Ideation, N = 73			Suicide Attempts, N = 22		
Demographic	N (%)	Yes, N (%)	No, N (%)	p-value	Yes, N (%)	No, N (%)	p-value
*Age (in years)*				0.017^&^			<0.001^&^
51–69	651 (23.1)	9 (12.3)	642 (23.4)		0 (0)	651 (23.3)	
41–50	630 (22.4)	14 (19.2)	616 (22.5)		0 (0)	630 (22.6)	
31–40	790 (28.1)	20 (27.4)	770 (28.1)		8 (36.4)	782 (28.0)	
18–30	745 (26.5)	30 (41.1)	715 (26.1)		14 (63.6)	731 (26.1)	
*Sex*				<0.001*			<0.001*
Male	1070 (38.0)	11 (15.1)	1059 (38.6)		0 (0)	1070 (38.3)	
Female	1746 (62.0)	62 (84.9)	1684 (61.4)		22 (100)	1724 (61.7)	
*Marital status*				0.134*			1.00*
Single^#^	541 (19.2)	19 (26.1)	522 (19.0)		4 (18.2)	537 (19.2)	
Married/cohabitating	2275 (80.8)	54 (73.9)	2221 (81.0)		18 (81.8)	2257 (80.8)	
**Socioeconomic**							
*Education level*				0.464*			0.508*
≥ Primary	1052 (37.4)	24 (32.9)	1028 (37.5)		10 (45.5)	1042 (37.3)	
No education	1763 (62.6)	49(67.1)	1714 (62.5)		12 (54.5)	1751 (62.7)	
Missing/not reported	1 (0.1)	0	1 (0.1)		0		
*Employment*				<0.014^&^			0.061^&^
Employed	479 (17.0)	12 (16.4)	467 (17.0)		2 (9.1)	477 (17.1)	
Self-employed	1556 (55.3)	30 (41.1)	1526 (55.6)		9 (40.9)	1547 (55.4)	
Unemployed/others	781 (27.7)	31 (42.5)	750 (27.3)		11 (50.0)	770 (27.5)	
*Household income*				0.334^&^			0.166^&^
Tertile 1 (low)	981 (34.5)	26 (35.6)	955 (34.8)		6 (27.2)	975 (34.9)	
Tertile 2 (medium)	843 (29.9)	26 (35.6)	817 (29.8)		4 (18.2)	839 (30.0)	
Tertile 3 (high)	910 (32.3)	18 (24.7)	892 (32.5)		11 (50.0)	899 (32.2)	
Missing/not reported	82 (2.9)	3 (4.1)	79 (2.9)		1 (4.6)	81 (2.9)	
*Place of residence*				0.249*			0.163*
Rural	1949 (69.2)	46 (63.0)	1903 (69.4)		12 (54.6)	1937 (69.3)	
Urban	867 (30.8)	27 (37.0)	840 (30.6)		10 (45.5)	857 (30.7)	
**Health and behaviour**							
*Alcohol use*				0.065^&^			0.157^&^
Non-drinker	1148 (40.8)	21 (28.8)	1127 (41.1)		4 (18.2)	1144 (40.9)	
Past drinker	309 (11.0)	6 (8.2)	303 (11.1)		3 (13.6)	306 (11.0)	
≤2 days per week	905 (32.1)	33 (45.2)	872 (31.8)		9 (40.9)	896 (32.1)	
>2 days per week	450 (16.0)	12 (16.4)	438 (15.9)		6 (27.3)	444 (15.9)	
Missing/not reported	4 (0.1)	1 (1.4)	3 (0.1)		0 (0)	4 (0.1)	
*Smoking*				0.544^&^			0.879^&^
Non-smoker	2177 (77.3)	57 (78.1)	2120 (77.3)		18 (81.8)	2159 (77.3)	
Current smoker	155 (5.5)	2 (2.7)	153 (5.6)		1 (4.6)	154 (5.5)	
Past smoker	484 (17.2)	14 (19.2)	470 (17.1)		3 (13.6)	481 (17.2)	
*Physical activity*				0.561*			0.279*
Yes	2522 (89.7)	64 (87.7)	2458 (89.6)		18 (81.8)	2504 (89.6)	
No	294 (10.4)	9 (12.3)	285 (10.4)		4 (18.2)	290 (10.4)	
*High blood sugar*				0.472*			0.638*
No	2537 (90.1)	66 (90.1)	2471 (90.1)		20 (90.9)	2517 (90.1)	
Yes	179 (6.4)	6 (8.2)	173 (6.3)		0 (0)	179 (6.4)	
Missing/not reported	100 (3.6)	1 (1.4)	99 (3.6)		2 (9.1)	98 (3.5)	
*High blood pressure*				0.332*			0.036*
No	1715 (67.1)	49 (67.1)	1666 (60.7)		17 (77.3)	1698 (60.8)	
Yes	1075 (38.2)	24 (32.9)	1051 (38.3)		3 (13.6)	1072 (38.4)	
Missing/not reported	26 (0.9)	0 (0)	26 (1.0)		2 (9.1)	24 (0.8)	
*Body mass index*				0.712*			0.823*
<25 kg/m^2^	1683 (59.8)	42 (57.5)	1641 (59.8)		12 (54.6)	1671 (59.8)	
≥25 kg/m^2^	1062 (37.7)	29 (39.7)	1033 (37.7)		9 (40.9)	1053 (37.7)	
Missing/not reported	71 (2.5)	2 (2.7)	69 (2.5)		1 (4.6)	70 (2.5)	
*Family suicide history*				0.002*			1.00*
No	2746 (97.5)	66 (90.4)	2680 (97.7)		22 (1000)	2724 (97.5)	
Yes	70 (2.5)	7 (9.6)	63 (2.3)		0 (0)	70 (2.5)	

^#^includes divorced, separated, and widowed

*Fisher’s exact test p-value

^&^Chi-square test p-value.

### Prevalence of suicidal ideation and suicide attempts

The weighted prevalence of suicidal ideation and suicide attempt was 3.1% and 0.7% respectively, in this study sample (Table 1). A majority of those reporting having suicidal ideation were females, those who were 18–30 years old, self-employed or unemployed, current drinkers, and did not have a family history of suicide. Most of the participants who reported attempting suicide were individuals aged 18–30 years, were unemployed, did not have high blood pressure, and those who attempted suicide were all females.

### Factors associated with suicidal ideation and suicide attempts

Based on the multivariable analyses, relative to individuals aged 51–69 years, those who were 18–30 (AOR: 4.59, 95% CI: 1.54–13.70) and 31–40 (AOR: 3.17, 95% CI: 1.31–7.66) years old had higher odds of suicidal ideation. Females (AOR: 3.92, 95% CI: 1.79–8.58), those who were single (AOR: 2.20, 95% CI: 1.22–3.98), unemployed (AOR: 2.01, 95% CI: 1.18–3.40), and reported of having family suicide history (AOR: 4.89, 95% CI: 1.72–13.96) were found to have higher odds of having suicidal ideation (Table 2). Compared to non-drinkers, those who currently consumed alcohol had higher odds of suicidal ideation, and the odds increased with the rising frequency of alcohol consumption. Those aged 18–30 years (AOR: 6.29, 95% CI: 1.85–21.38) compared to those >30 years old, and who currently drink alcohol (AOR: 4.93, 95% CI: 1.09–22.45) had higher odds of suicide attempts. Furthermore, compared to those in the highest income group, the odds of suicidal ideation for participants from the middle and lowest income group increased by 2.39 (95% CI: 1.17–4.87) and 3.06 (95% CI: 1.40–6.68) times higher, however, the odds of attempting suicide for those in the middle-income group reduced by 77% (AOR: 0.23, 95% CI: 0.06–0.84).

**Table 2:** Factors associated with suicidal ideation and suicide attempts in Bhutan (unadjusted and adjusted odds ratio) (N = 2,816).

Variables	Suicidal Ideation		Suicide Attempt	
	COR (95% CI)	AOR (95% CI)^1^	COR (95% CI)	AOR (95% CI)^2^
**Demographic**				
*Age (ref*: *51–69)*				
41–50	1.83 (0.64–5.24)	3.06 (0.98–9.52)		
31–40	1.81 (0.75–4.37)	**3.17 (1.31–7.66)**		
18–30	**3.48 (1.27–9.52)**	**4.59 (1.54–13.70)**	6.01 (2.17–16.66)^*^	6.29 (1.85–21.38)^*^
*Sex (ref*: *Male)*				
Female	**4.96 (2.30–10.68)**	**3.92 (1.79–8.58)**	NA	
*Marital status (ref*: *Married/ cohabitating)*				
Single^#^	**1.74 (1.04–2.90)**	**2.20 (1.22–3.98)**	1.40 (0.38–5.08)	
**Socioeconomic**				
*Education level (ref*:>Primary)				
No education	1.31 (0.68–2.53)		0.73 (0.25–2.18)	
*Employment (ref*: *Employed/self-employed)*				
Unemployed/others	**3.10 (2.08–4.61)**	**2.01 (1.18–3.40)**	**2.93 (1.17–7.34)**	
*Household income (ref*: High*)*				
Medium	1.62 (0.83–3.18)	**2.39 (1.17–4.87)**	**0.20 (0.06–0.72)**	**0.23 (0.06–0.84)**
*Low*	1.82 (0.92–3.63)	**3.06 (1.40–6.68)**	0.64 (0.17–2.35)	0.91 (0.22–3.92)
*Place of residence (ref*: *Rural)*				
Urban	1.05 (0.56–1.94)		2.20 (0.78–6.20)	
**Health and behaviour**				
*Alcohol use (ref*: Non-drinker*)*				
Past drinker	0.54 (0.16–1.82)	1.09 (0.34–3.55)		
≤2 days per week	1.68 (0.93–3.05)	**2.81 (1.44–5.48)**		
>2 days per week	1.18 (0.47–2.98)	**3.43 (1.23–9.54)**		
*Alcohol use (ref*: *Non-drinker)*^*§*^				
Past drinker			2.34 (0.43–12.59)	4.72 (0.72–31.07)
Current drinker			2.92 (0.79–10.78)	**4.93 (1.09–22.45)**
*Smoking (ref*: *Non-smoker)*				
Current smoker	0.56 (0.13–2.44)		1.49 (0.21–10.43)	
Past smoker	0.67 (0.32–1.41)		0.57 (0.16–2.01)	
*Physical activity (ref*: *Yes)*				
No	0.99 (0.43–2.30)		3.01 (0.82–11.07)	
*High blood sugar (ref*: *No)*				
Yes	1.46 (0.54–3.99)		NA	
*High blood pressure (ref*: *No)*				
Yes	0.83 (0.45–1.54)		0.43 (0.09–2.13)	
*Body mass index (ref*: *<25* kg/m^2^*)*				
≥25 kg/m^2^	0.82 (0.42–1.59)		1.09 (0.35–3.42)	
*Family suicide history (ref*: *No)*				
Yes	**3.73 (1.44–0.69)**	**4.89 (1.72–13.96)**	NA	

COR: crude odds ratio; AOR: Adjusted odds ratio; Bold: significant at 5% level

^#^includes divorced, separated, and widowed

*the reference group for this estimate is 31–69 years

^§^used in the regression models for Suicide Attempt

^1^ Models adjusted for age, sex, marital status, employment, household income, alcohol use, and family history of suicide

^2^ Models adjusted for age, employment, household income, place of residence, alcohol use, and physical activity.

## Discussion

Using the nation-wide 2014 Bhutan STEPS Survey dataset, we examined the risk factors associated with suicidal ideation and suicide attempts. Around 3% of the adults aged 18–69 years reported having suicidal ideation, and 0.7% ever attempted suicide. The prevalence is slightly higher than that of the twelve-month global estimate of 2.1% for suicidal ideation and 0.4% for suicide attempts in developing countries [8]. However, the occurrence of ever suicide attempts is lower than the global lifetime estimate of 2.7% [5]. The prevalence is marginally lower than that of the 2015 Bhutan GNH Survey. The possible reason could be that in addition to adults, the GNH Survey also recruited participants <18 years of age, among which the literature has reported high rates of suicidal thoughts and behaviour. In this study, we found female gender, being single, being unemployed, low and middle household income, and having a family history of suicide were independently associated with having suicidal ideation. Younger age and alcohol consumption were associated with both suicidal ideation and suicide attempts. Whereas, those from the middle-income group had reduced odds of attempting suicide. The findings can be useful in informing targeted policy investments aimed to prevent suicide in Bhutan.

We found that females were more likely to have suicidal ideation and attempted suicide than males. Our finding aligns with the data in other studies [3, 4, 10–12, 21] and also supports the results in an earlier study from Bhutan [23]. Females may be more likely to feel sad, helpless, and hopeless, and such mental conditions are associated with suicidal thoughts and behaviour [3, 15]. Literature suggests that Bhutanese females are also more likely to experience poor mental health than men [27, 36]. Mental health and socioeconomic factors have been shown to explain a large portion of the higher odds of suicidal attempts among girls than boys [21]. Social and cultural context specific to a country concerning the status of girls and women in society may help elucidate the gender differences in suicidal thoughts and behaviour. Although the role of women in society and development is increasingly being recognized, traditionally Bhutan was and is still a patriarchal society. Owing to male dominance, the issues women and girls are facing might have received less attention that in turn, could adversely impact their mental health. The finding that those who were single had higher odds of suicidal ideation is in agreement with data in the literature [5, 8]. Divorced or separated Bhutanese also had an increased risk of having mental conditions [36].

Consistent with findings in some studies including the study that analysed the Bhutan GNH Survey data [3, 9–11, 23], our results also showed that younger adults had higher odds of attempting suicide and suicidal ideation. Young individuals, especially adolescents, may experience higher levels of stress and emotional disorders, have greater alcohol and illicit drug use, and risk-taking behaviours [3]. They may be vulnerable to negative social cues and transmission of adverse behaviours [3]. In Bhutan, the prevalence of alcohol (24.2%) and substance abuse (16%), and mental health markers such as being physically attacked (39%), bullying (27%), and loneliness among adolescents were found to be high [28]. Our results also revealed that those with a family history of suicide had higher odds of suicidal ideation. Studies indicate that family history of suicidal behaviour is associated with the risk of suicide and multiple suicide attempts independent of mental illness and other factors [7, 16–18]. Impulsivity and aggression have been shown to mediate the association between family suicide history and suicide [18]. Poor problem-solving and coping abilities have also been posited to be some pathways through which family suicide history may influence suicide [17, 18]. Besides, genetic factors have been thought to be more important than non-genetic factors for the familial clustering of suicide [16].

Furthermore, our study revealed that those who consumed alcohol had higher odds of having both suicidal ideation and suicide attempts, and the odds of suicidal ideation increased with higher frequency of consumption. There is adequate data which indicate that alcohol use disorder significantly increases the risk of suicidal thoughts and behaviours, including suicide [3, 11, 19]. In 2016, the harmful use of alcohol was responsible for more than 3 million deaths annually [37]. The health and socioeconomic burdens attributable to alcohol consumption are mainly from injuries, violence, and suicides. The WHO estimates that alcohol is accountable for 18% of the suicides and another 18% of interpersonal violence [37]. In Bhutan, alcohol consumption is socially accepted, and the prevalence of alcohol consumption is very high at 42.4%, with more than 22% engaging in heavy episodic drinking [26]. Harmful alcohol use is the top leading cause of mortality in Bhutan [34] and has been related to numerous major social and health issues such as alcoholic liver disease, injuries, violence, crime, including suicide [29, 38].

We also found that those in the lowest and the middle-income group had greater odds of suicidal ideation. Similarly, unemployed individuals had higher odds of suicidal ideation and suicide attempts (in the bivariate analysis). Data suggest that those who are socioeconomically disadvantaged are at higher risk of suicidal behaviours [5, 8, 10]. People from wealthier households may have better access to financial resources and social support systems that can enable them to lead a healthy life both physically and mentally than those with low socioeconomic background. The 2014 Bhutan Suicide Study also showed that a majority of those who attempted suicide were from the low socioeconomic background [29]. Another study that analysed the Bhutan GNH Survey data also found that those with low income and illiterate had higher odds of common mental disorders [36]. Conversely, our results showed that those in the middle-income group had lower odds of attempting suicide than those in the high-income group. The influence of income on suicidal attempt warrants further investigation with better data. Nevertheless, socioeconomic factors such as income, employment, and education did not emerge to significantly predict suicidal ideation and attempt in the study that analysed the 2015 GNH Survey data [23]. This may suggest that physical, behavioural, and mental health factors might be more important than socioeconomic factors.

### Strengths and limitations

This study used a large sample data from a nationally-representative survey that used the WHO-recommended approach to monitor NCD risk factors. The survey instruments were validated, and trained surveyors collected the data. The survey achieved a very high response rate (97%). All of these lends credibility to the results in our study. Besides, our study employed the complex survey data analysis to account for the complex survey design, clustering, and sample weights of the Bhutan STEPS Survey. Therefore, the findings in this study can be widely applicable to the Bhutanese population. We also examined the role of some important factors such as alcohol consumption, BMI, family history of suicide, and smoking status that were not investigated in an earlier study from Bhutan.

There are, however, some limitations to this study. First, reporting of suicide information can be very sensitive due to the associated social stigma that could have led to underreporting of suicide information [3, 25]. Thus, the prevalence of suicidal ideation and suicide attempt may be underestimated. Likewise, the Bhutan STEPS Survey did not include individuals <18 years of age and studies including our current study found high rates of suicidal ideation and attempt among adolescents and young adults [3, 9, 11, 28]. For a more significant public health gain, future studies may consider monitoring suicidal ideation among adolescents. Owing to non-availability of information, we could not examine the influence of important factors such as mental illness, social environment, academic pressure, illicit drug use, physical abuse, sexual intercourse, and parental separation found to be predictors of suicidal thoughts and behaviours [3, 4, 9, 11, 12, 21, 23]. Some of these factors, including domestic violence and marital problems, have also been shown to be possible contributing factors for suicide in Bhutan [29]. Future studies with better designs, therefore, need to consider these factors. Finally, given its cross-sectional design, causal inferences cannot be made for the associations found in this study.

## Conclusions

Based on the 2014 Bhutan STEPS Survey data, the prevalence of suicidal ideation and suicide attempts in Bhutan was 3.1% and 0.7%, respectively. The risk factors influencing suicidal ideation and suicide attempt identified in this study were younger age, female gender, low income, family history of suicide, alcohol consumption, and unemployment. The findings can be useful for policymakers in designing appropriate strategies for the prevention of suicidal behaviours. Suicide prevention interventions that target young people and females and those in the low socioeconomic groups may be cost-effective. Prevention measure such as screening programs to identify those at risk, restricting access to means, awareness and stigma reduction programs, and enhancing access to quality mental health services needs to be scaled-up. Intensified efforts to reduce and control unhealthy behaviours, especially harmful use of alcohol may help prevent suicide.
